# Loneliness among Older Adults in an Intergenerational Telephone-Based Program

**DOI:** 10.1093/geroni/igaf122.2782

**Published:** 2025-12-31

**Authors:** Rebecca Mullen, Joanna Fitzgibbons, Prajakta Shanbhag, Mahi Mahfuza Haque, Sarah Tietz, Devin Gilhuly, Jodi Waterhouse

**Affiliations:** University of Colorado School of Medicine, Aurora, Colorado, United States; University of Colorado Anschutz Medical Campus, Aurora, Colorado, United States; University of Colorado School of Medicine, Aurora, Colorado, United States; University of Colorado School of Medicine, Aurora, Colorado, United States; University of Colorado School of Medicine, Aurora, Colorado, United States; University of Colorado School of Medicine, Aurora, Colorado, United States; CU Anschutz Medical Campus, Aurora, Colorado, United States

## Abstract

Loneliness is a critical health concern for older adults, yet few evidence-based interventions for loneliness exist. Connecting Older Adults to Students Through Intergenerational Telecare (COAST-IT) is a telephone-based program that pairs health professional students from the University of Colorado Anschutz Medical Campus with community-dwelling older adults for unstructured conversations. This study assessed the prevalence, characteristics, and changes in loneliness in COAST-IT older adult participants. We conducted cross-sectional surveys of older adult participants at pre-intervention and post-intervention (n = 38). Loneliness was measured using the UCLA 3-item Loneliness Survey. We also conducted multivariable regression controlling for age, sex, marital status, sexual identity, duration in study, and communication frequency with student partner. The mean age of participants was 74.1 (SD = 6.98), with the majority being female (89.5%, 34/38) and White (92.1%, 35/38). The average length in the study was 7.86 months (SD = 4.52). Nearly half (47.4%, 18/38) of older adult participants communicated with their student partner less than twice per month, while 44.7% (17/38) did so more often. The prevalence of loneliness at baseline was 29.7% (11/38), compared to 18.4% (7/38) at follow-up (p = 0.41). After adjusting for covariates, loneliness was significantly associated with older age (p < 0.001), being divorced (p = 0.009), and sexual minority status (p = 0.002). In conclusion, older adults who participated in an intergenerational telephone-based program with students did not experience a significant reduction in loneliness. Future research will examine the program’s impact on older adult participants’ depression and anxiety symptoms in addition to student participants’ loneliness and ageist beliefs.

